# The differential effect of resveratrol on the renal artery of normal and diabetic rats

**DOI:** 10.1186/2050-6511-13-S1-A48

**Published:** 2012-09-17

**Authors:** Ljiljana C Gojković-Bukarica, Vladimir I Kanjuh, Radmila B Novaković, Dragana D Protić, Jelena M Cvejić, Milica T Atanacković

**Affiliations:** 1 Institute of Pharmacology, Clinical Pharmacology and Toxicology, Medical Faculty, University of Belgrade, 11129 Belgrade, Serbia; 2 Serbian Academy of Sciences and Arts, 11129 Belgrade, Serbia; 3 Department of Pharmacy, Faculty of Medicine, 21000 Novi Sad, Serbia

## Background

Resveratrol, a polyphenol present in red wine, is thought to be responsible for cardiovascular benefits associated with moderate wine consumption. The mechanisms by which resveratrol causes vasodilatation are uncertain. The aim of this study was to investigate the mechanisms of resveratrol-induced vasorelaxation of rat renal artery (RA) with endothelium in normal and diabetic rats.

## Methods

Alloxan was used for the induction of diabetes in rats. Samples of RA were obtained from male Wistar rats and were mounted in an organ bath for recording isometric tension. The experiments followed a multiple curve design.

## Results

Resveratrol relaxed RA of normal rats more potently than RA of rats with diabetes (EC_50_ 8 and 50 µM, respectively). L-NAME and methylene blue partly antagonized the relaxation of RA of normal animals only. A nonselective blocker of voltage-gated K^+^ (K_V_) channels, 4-aminopyridine (4-AP) partly inhibited the relaxation of RA of normal as well as of diabetic rats. However, margatoxin, a selective antagonist of K_V_1.x channels, completely antagonized the relaxation of RA of diabetic rats only. Glibenclamide, a highly selective blocker of ATP-sensitive K^+^ channels, did not block resveratrol-induced relaxation in both experimental models.

## Conclusions

In conclusion, we have shown that resveratrol induces a strong endothelium-dependent relaxation of RA of normal rats, and that 4-AP-sensitive K^+^ channels are involved in this relaxation. In diabetic rats, resveratrol induced NO-independent relaxation and maragtoxin-sensitive K^+^ channels are involved.

